# New Method of Mounting an Artificial Denture

**Published:** 1887-04

**Authors:** H. W. Howe


ARTICLE VI.

### NEW METHOD OF MOUNTING AN ARTIFICIAL DENTURE.

BY H. W. HOWE, D. D. S.

Without entering into the discussion of the relative values of the different bases for artificial dentures, we assume that an incorruptible metallic one is the best. The two metals most acceptable are gold and platinum, and while the platinum plate with its continuous gum is the most artistic, still a case can be mounted on gold by a somewhat new method, which has all the merits of continuous gum except the gum color. It is a description of this process to which I invite your attention.

The impression casts and dies are obtained in the usual way, and after the plate has been swaged and articulation taken, the teeth, which must be of the recently introduced countersunk pattern, are arranged in their desired position and wax built about them in such a manner as shall best restore the contour of the face. After the teeth are in position and the wax well smoothed to the shape wanted in the finished case, carefully remove each tooth, partially fill up the depressions left in the wax, leaving sufficient rim to show where each tooth belongs. Now varnish the whole, and when dry take an impression of the palatine surface in sand; make dies of this surface, and stamp gold plate of same gauge as the original plate. This piece of gold will cover the wax on the palatine surface. After filing that portion of the rim that comes next the teeth to correspond to their interstices, the impression material is removed and the rim and plate soldered together; the holding of the two together can be greatly facilitated by riveting with fine gold wire, which after the soldering can be filed away. There is little danger of the plate warping, if it is heated uniformly with a blast blaze. It should, in soldering, rest steadily on a piece of charcoal cut to fit it. After the soldering, the plate is filed and polished, and then replaced upon the articulation. Each tooth is now fitted to its position and re-articulated. The case is then flasked teeth down, exposing only a small portion of the wax rim. The procedure is now the same as in rubber work, the wax is boiled out and the space packed with pink rubber. Much artistic talent may now be displayed in the carving of the gum. After polishing and bleaching, the case is finished. It will rival continous gum for beauty, while it is far stronger and more serviceable. A broken tooth is easily replaced in oxy-phosphate of zinc, in ten minutes.

The writer offers this method to the profession, believing it a substantial improvement on what has heretofore been attempted in gold work, and hopes it will meet with their approbation.—*Western Dental Journal.*

---

ARTICLE VII.

